# CNS-Sparing Histamine H_3_ Receptor Antagonist as a Candidate to Prevent the Diabetes-Associated Gastrointestinal Symptoms

**DOI:** 10.3390/biom12020184

**Published:** 2022-01-22

**Authors:** Arianna Carolina Rosa, Patrizia Nardini, Silvia Sgambellone, Maura Gurrieri, Simona Federica Spampinato, Alfonso Dell’Accio, Paul L Chazot, Ilona Obara, Wai L Liu, Alessandro Pini

**Affiliations:** 1 Department of Scienza e Tecnologia del Farmaco, University of Turin, Via P. Giuria 9, 10125 Turin, Italy; simonafederica.spampinato@unito.it; 2 Department of Clinical and Experimental Medicine, University of Florence, Viale Pieraccini 6, 50139 Florence, Italy; patrizia.nardini@unifi.it (P.N.); maura.gurrieri@live.it (M.G.); alfonso.dellaccio@unifi.it (A.D.); 3 Department of Neuroscience, Psychology, Drug Research and Child Health (NEUROFARBA), Pharmacology and Toxicology Section, University of Florence, Viale Pieraccini 6, 50139 Florence, Italy; silvia.sgambellone@unifi.it; 4 School of Biological and Biomedical Science, Durham University, Durham DH1 3LE, UK; paul.chazot@durham.ac.uk; 5 School of Pharmacy and Translational and Clinical Research Institute, King George VI Building, Newcastle University, Newcastle-upon-Tyne NE1 7RU, UK; ilona.obara@newcastle.ac.uk; 6 Liu & Co Consulting Limited, Whitstable CT5 3RF, UK; steve.liu08@hotmail.co.uk

**Keywords:** histamine, PF0086087, ZPL-868, diabetes, gastrointestinal neuropathy

## Abstract

Among the histamine receptors, growing evidence points to the histamine H_3_ receptor as a pharmacological candidate to counteract the autonomic neuropathy associated with diabetes. The study aimed to evaluate the effect of PF00868087 (also known as ZPL-868), a CNS-sparing histamine H_3_ receptor antagonist, on the autonomic neuropathy of the intestinal tract associated with diabetes. Diabetes was induced in male BALB/c mice by a single high dose of streptozotocin (150 mg/kg). Colorectal specimens from control and diabetic mice, randomized to vehicle or PF0086087 (10, 30, 100 mg/kg/day by oral gavage for 14 days), were processed for morphological and immunohistochemical analysis. A significant overproduction of mucus in the intestinal mucosa of diabetic mice compared to the controls was observed. PF0086087 at the highest dose prevented mucin overproduction. The immunohistochemistry analysis demonstrated that diabetes causes a decrease in the inhibitory component of enteric motility, measured as the percentage of neuronal nitric oxide synthase-positive neurons (*p* < 0.05) and a parallel increase in the excitatory component evaluated as substance P-positive fibres (*p* < 0.01). PF0086087 dose-dependently prevented these pathophysiological events. In conclusion, PF0086087 may be an essential tool in preventing nitrergic dysfunction in the myenteric plexus of the distal colon and diabetes-induced gastrointestinal complications.

## 1. Introduction

Gastrointestinal (GI) neuropathy, leading to complications such as gastroesophageal reflux disease (GERD), gastroparesis, diarrhoea, habitual constipation and faecal incontinence, is one of the microvascular complications associated with diabetes [1,2]. Many pathways are involved in developing the clinical GI symptoms in diabetes [3]. The excessive glucose concentration in the blood causes the production of advanced glycation end products (AGEs), which promote, in turn, neuronal damage. The reduction in the number of neurons of the central and autonomic nervous systems (CNS and ANS, respectively) and the interstitial cells of Cajal (ICC), the physiological gut pacemakers [4], generates a dysregulation of motility, accompanied by damage to smooth muscle cells and decreased contractility [3]. AGEs have been reported to activate mast cells and may contribute to a vicious cycle increasing the formation of AGEs itself [5] and promoting neurogenic inflammation [6]. Histamine may have an active role in the establishment of this vicious circle. Binding to its receptor RAGE on mast cells, AGEs induce histamine exocytosis and the production of reactive oxygen species (ROS). ROS participates in a feedback loop on AGE production [5], while histamine activates histamine-sensitive fibres, generating an orthodromic action potential in which substance P (SP) and other neurotransmitters are released with consequent further mast cell degranulation [6].

At the hypogastric ganglion level, Atencio et al. (2020) hypothesized that SP mediates the vicious circle between histamine and the noradrenergic sympathetic response via noradrenaline release [7]. Among the four histamine receptor subtypes, numerous reports demonstrated the presence of presynaptic histamine H_3_ receptors in the autonomic nervous system. These heteroreceptors negatively control the release of several neurotransmitters, including acetylcholine, dopamine, noradrenaline, and serotonin in the GI tract [8,9,10,11]. Furthermore, histamine H_3_ receptors act as autoreceptors and negatively affect histamine release itself [12,13,14]. Therefore, the presynaptic histamine H_3_ receptor could be crucial in regulating the peripheral sympathetic reflex [7]. Consistently, Silver et al. (2001) demonstrated that activation of histamine H_3_ receptors in the peripheral sympathetic terminal inhibits the Na^+^/H^+^ exchanger (NHE) activity, thus reducing the noradrenaline release during myocardial ischemia [15]. Targeting the presynaptic histamine H_3_ receptor could represent an intriguing strategy to counteract diabetic autonomic neuropathy. However, the activity of histamine H_3_ receptors appears to differ according to their central and peripheral distribution. In pain modulation, for example, when histamine is injected directly into various brain areas, it attenuated pain [16,17]; on the contrary, in the peripheral nervous system, histamine is released in response to tissue injury/damage and contributes to the generation of pain hypersensitivity [12,18]. The role of the histamine H_3_ receptor is controversial also in diabetes, with both histamine H_3_ receptor agonism [19,20] and inverse agonism, via pitolisant [21], demonstrating improved glucose tolerance in obese mice. Consistently, histamine H_3_ receptor-deficient mice displayed a metabolic syndrome characterized by obesity, hyperphagia, and increased leptin and insulin levels [19,22].

The CNS-sparing histamine H_3_ receptor antagonist, 4-(5-([1,4′-bipiperidin]-1′- yl)-1,3,4-thiadiazol-2-yl)-2-(pyridin-2-yl)morpholine (PF00868087, also known as ZPL-868) [23], was initially developed and tested for the treatment of allergic rhinitis [24]. Interestingly, PF00868087 also showed promising antidiabetic effects [21]. Herein, we decided to evaluate the effect of PF00868087 on the autonomic neuropathy of the intestinal tract associated with diabetes in a mouse model of short-term diabetes, induced by a single high-dose (150 mg/kg i.p.) streptozotocin (STZ) injection, previously shown to induce a robust and early neuropathic phenotype [25].

## 2. Materials and Methods

### 2.1. Animals

Six-week-old male BALB/c mice were maintained in compliance with the European Council directives (No. 2010/63/EU) and with the Principles of Laboratory Animal Care (NIH No. 85-23, revised 2011). The animals were kept at constant environmental and nutritional conditions at 25 ± 2 °C, with alternating 12 h light and dark cycles, and fed a standard diet during a 5-day adaptation period. They were fed a standard pellet diet (Piccioni, Settimo Milanese, Milan, Italy) and watered ad libitum. The scientific project was approved by the Ethical Committee of Florence University and the Italian Ministry of Health (Authorization N. 192/2017).

### 2.2. Experimental Protocol

Diabetes was induced by a single dose of STZ (150 mg/kg i.p.). Diabetes was defined as a fasting blood glucose level ≥200 mg/dL, and the onset of diabetes was evaluated by measuring 6 h fasting blood glucose using a Glucocard MX Blood Glucose Meter. After the onset of diabetes, PF0086087, CNS-sparing histamine H_3_ receptor antagonist, was administered daily for 14 days by oral gavage at 10, 30, 100 mg/kg. Weight, food, and water intake were recorded daily. At the end of the experimental period, mice were sacrificed, and distal colon specimens were collected for morphological analysis. The distal colon was quickly removed from the abdomen, washed with ice-cold physiological saline solution, and fixed in 4% paraformaldehyde in 0.1 M phosphate-buffered saline (PBS) pH 7.4. After embedding in paraffin, full-thickness cross-sections (5 µm thick) were cut and used for morphological analysis.

### 2.3. Histological Staining

Haematoxylin and eosin (H/E) staining and periodic acid-Shiff (PAS) reaction were performed in a single session to minimize artefactual staining differences, and at least three sections per animal were analysed. H/E staining was used for evaluation of the distal colon morphology, whilst PAS reaction was used for semi-quantitative morphometric analysis of the mucins. Analyses were carried out acquiring at 20× and 40× objectives, of at least 10 regions of interest (ROIs) randomly taken for each section with a microscope equipped with a camera (Leica DFC310 F× 1.4-megapixel camera, Leica Microsystems, Mannheim, Germany). Histological assessment of the submucosal oedema was performed, measuring the space interposed between the mucosa and muscularis propria, as previously described [26]. PAS-positive area and intensity were measured by the ImageJ software (NIH, Bethesda, ML, USA), and reported as integrated density (mean grey value*positive area/total area of ROI).

### 2.4. Immunohistochemistry

For immunofluorescence analysis, rehydrated sections were submerged in Tris buffer (10 mM) with EDTA (1 mM, pH 9.0) for 20 min at 90–92 °C for antigen retrieval. The sections were then washed in PBS, blocked with 1.5% bovine serum albumin (BSA, Applichem, Darmstadt, Germany) in PBS to minimize non-specific binding and incubated overnight at 4 °C with primary antibodies (Table 1).

On the following day, the sections were incubated for 2 h at RT with appropriate fluorochrome-conjugated secondary antibodies diluted in BSA 0.15% PBS. The omission of the primary antibodies was used as the negative control. Sequential staining of the two antibodies, protein gene product 9.5 (PGP9.5) and neuronal nitric oxide synthase (nNOS), was performed for the double labelling reactions. Subsequently, the specimens were rinsed with PBS and mounted with an aqueous medium (FluoroshieldTM with DAPI, Thermo Fisher Scientific). The immunolabeled sections were observed under an epi-fluorescence Olympus BX40 microscope coupled to analySIS∧B Imaging Software (Olympus, Milan, Italy) using excitation filters for Alexa 594 red and Alexa 488 green with 20× and 40× objectives.

The total number of PGP9.5- and nNOS-immunoreactive cells was evaluated within the myenteric ganglia along the entire section by two independent observers (A.P., P.N.) blind to each other, and the results are expressed as the ratio of nNOS and PGP9.5 positive neurons per sections ± S.E.M (at least three sections per animal). The quantification of SP and vasoactive intestinal peptide (VIP) positive structures (nerve fibres) was morphometrically assessed within the myenteric ganglia on digitized images acquired with 40× objective using the threshold tool of ImageJ software (at least 3 sections/animal). The results are expressed as the ratio between the VIP positive area and the total area of myenteric ganglia considered in the analysis. 

### 2.5. Data Analysis and Statistical Tests

The data are expressed as the mean ± S.E.M. of seven animals per group. Statistical analysis was performed using GraphPad Prism 9.0 software (GraphPad, San Diego, CA, USA). The analysis of variance (one-way ANOVA) followed by Newman–Keuls was carried out to compare the groups, and a *p*-value ≤ 0.05 was considered significant.

When the data were not representative of a normal distribution, the non-parametric Kruskal–Wallis test was performed.

## 3. Results

**Figure 1:**
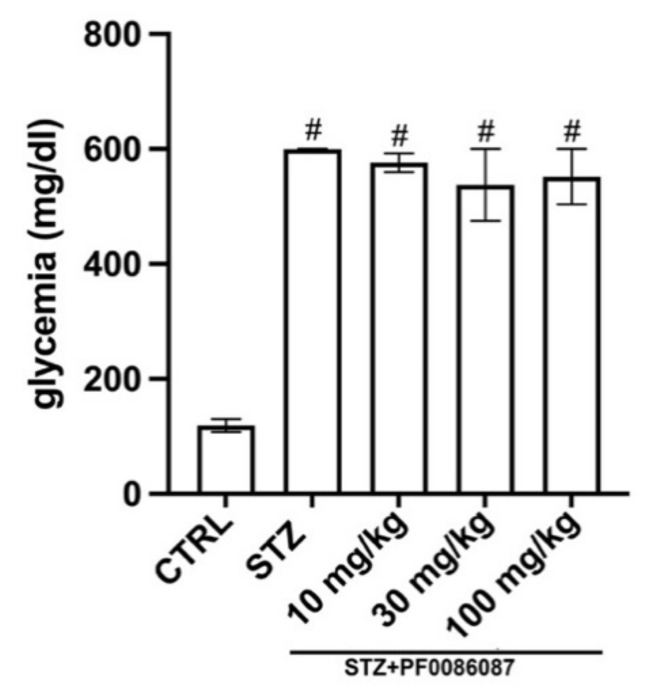
Effect of PF0086087 on glycaemic status. The STZ-induced mice showed severe hyperglycaemia. PF0086087 administration was not able to prevent hyperglycaemic status. One-way ANOVA test, the significance of difference # *p* < 0.01 vs. CTRL.

**Figure 2:**
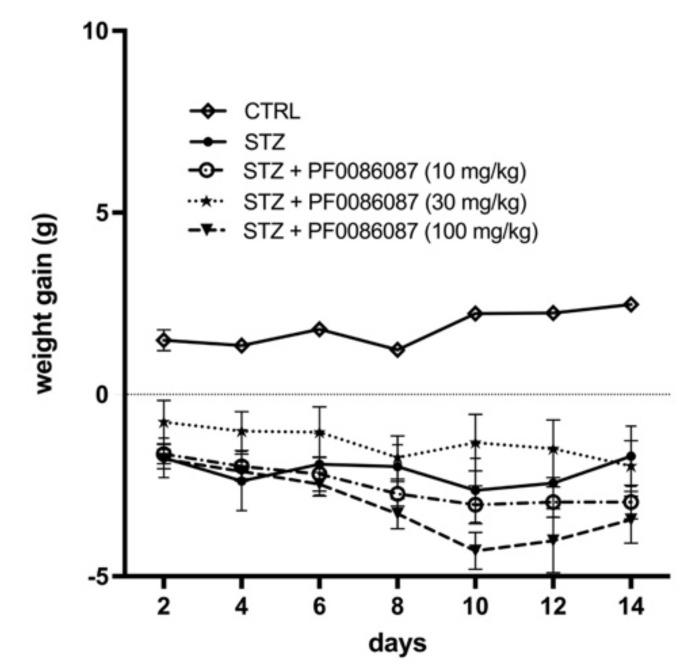
Effect of PF0086087 on body weight gain. Body weight was monitored daily from diabetes development, and weight gain was calculated. The one-way ANOVA test was applied. No significant differences were found among the STZ-induced mice of the different experimental groups.

Three days after STZ injection, all mice developed diabetic status (≥200 mg/dL), measured by the 6 h fasting glycaemia. At the end of experimental period, a severe hyperglycaemia was reached in STZ group (599 ± 2 mg/dL vs. 119 ± 11 mg/dL of controls), accompanied by a significant weight loss; PF0086087 administration did not affect hyperglycaemic status (576 ± 17 mg/dL, 537 ± 62 mg/dL, 552 ± 48 mg/dL, respectively) (Figure 1), nor body weight gain (Figure 2).

### 3.1. Effects of PF0086087 on the Distal Colon Morphology

**Figure 3:**
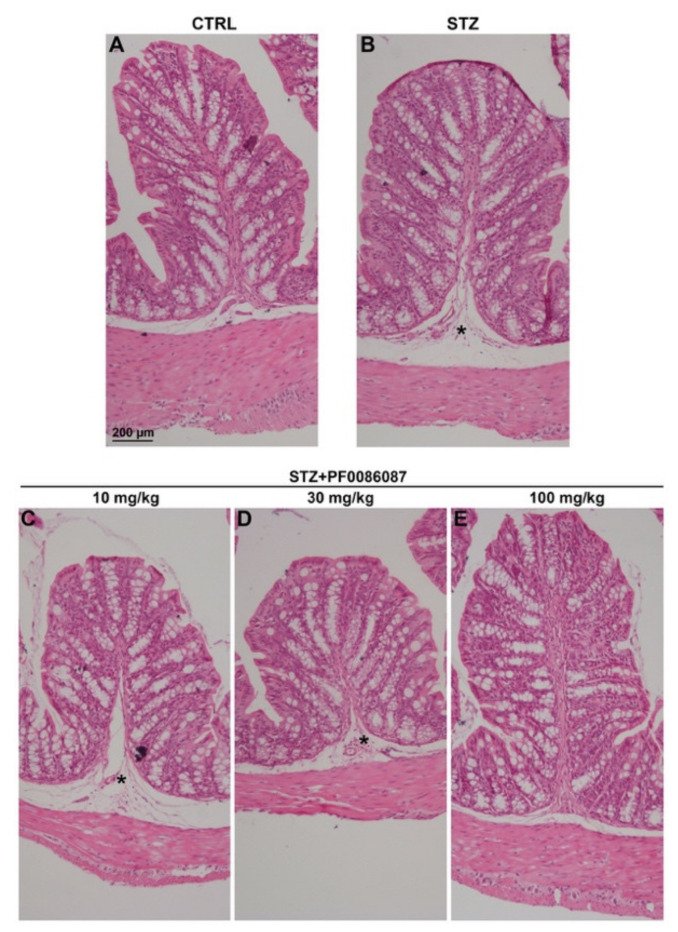
H/E staining of the mouse distal colon. The general morphology of the distal colon was preserved in all animals of the different experimental groups (**A**–**E**), with no relevant signs of degeneration, except for oedema in the submucosa of STZ-induced mice (asterisk). Scale bar = 200 µm.

**Figure 4:**
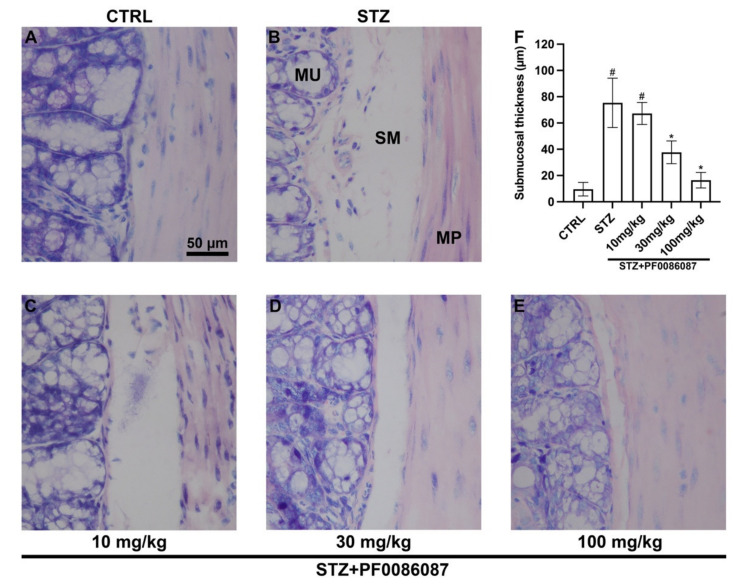
Histological assessment of submucosal oedema. The evaluation of oedema in the colon submucosal layer was performed measuring the space interposed between the mucosa and muscularis propria. Morphometrical analysis revealed increased oedema in STZ-induced mice compared with control (**A**,**B**,**F**). PF0086087 administration at the highest doses significantly reduced the oedema (**D**,**E**,**F**). One-way ANOVA test, significance of difference # *p* < 0.01 vs. CTRL; * *p* < 0.05 vs. STZ. MU, mucosa. SM, submucosa. MP, muscularis propria. Scale bar = 50 µm.

The effect of short-term STZ-induced hyperglycaemia on the anatomical structure of the distal colon was examined using morphological techniques. The H/E staining performed on the descending colon mucosa of the induced mice revealed folded mucosal villi, regular inter-cryptic distances and an almost continuous lining epithelium (Figure 3). In contrast, histological assessment of the submucosal layer revealed the presence of oedema in STZ-induced mice compared with control (Figure 3, asterisks and Figure 4). PF0086087 administration significantly reduced oedema in the colon submucosa in a dose-dependent manner (Figure 4).

**Figure 5:**
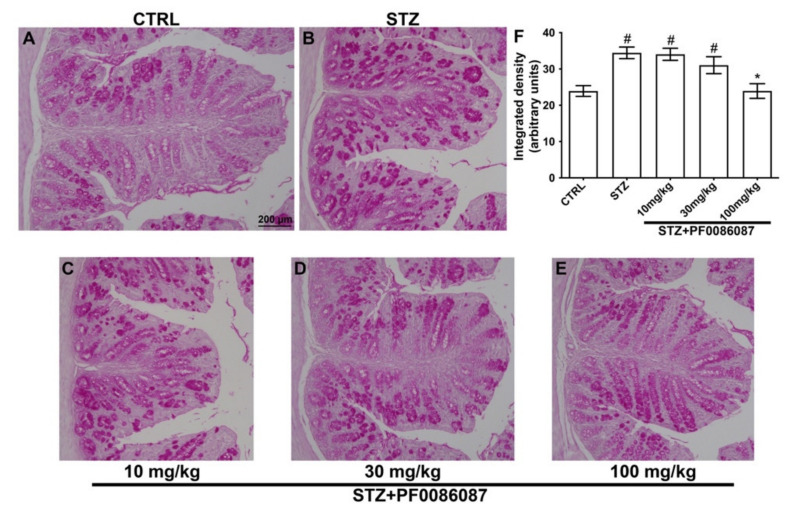
PAS reaction on the mouse distal colon. (**A**–**E**): representative micrographs at 20× magnification of PAS-stained distal colon sections. Scale bar = 200 µm. F: densitometric analysis of PAS reaction. Kruskal–Wallis test, significance of difference # *p* < 0.05 vs. CTRL; * *p* < 0.05 vs. STZ. Semi-quantitative analysis revealed that STZ significantly increased PAS staining area, indicating mucin overproduction. PF0086087 administration at the highest dose restored mucin synthesis to the level of control (**A**,**E**,**F**).

The expression of mucins was also evaluated using the PAS reaction. Surprisingly, the semi-quantitative analysis of PAS staining revealed increased production of mucins in the STZ-induced animals in respect to controls (Figure 5, panels A, B and F), at least partly explainable by a goblet cell hyperplasia in response to colon dysmotility. Once again, the treatment with PF0086087 at the highest dose suppressed mucin overproduction (Figure 5, panels B, E and F).

### 3.2. Effects of PF0086087 on the STZ-Induced Alteration of the Myenteric Plexus Neurons

**Figure 6:**
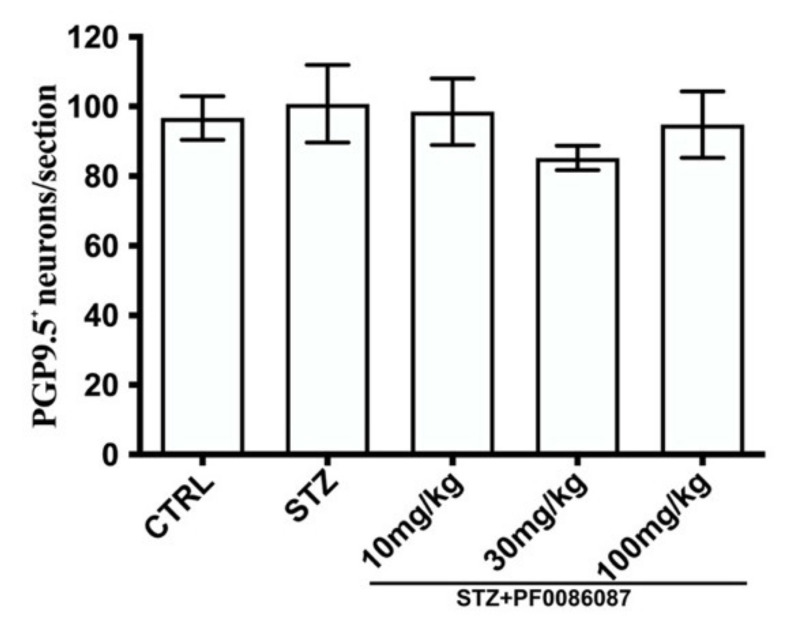
Quantification of PGP9.5 positive neurons. The pan-neuronal marker, PGP 9.5, was used to count the neuronal bodies per section. The one-way ANOVA test was used. No significant difference was found among the experimental groups.

The impact of STZ-induced hyperglycaemia upon the myenteric neuronal population in the distal colon was also evaluated. PGP 9.5, a pan-neuronal marker, was used to identify and count the neuronal cell bodies. No significant difference was found in the total number of PGP9.5 positive neurons among the experimental groups (Figure 6).

However, specific differences were revealed while evaluating the neuronal sub-populations of the myenteric plexus. The nNOS-positive neurons were counted in the myenteric ganglia, and the nNOS/PGP9.5 percentage was calculated.

**Figure 7:**
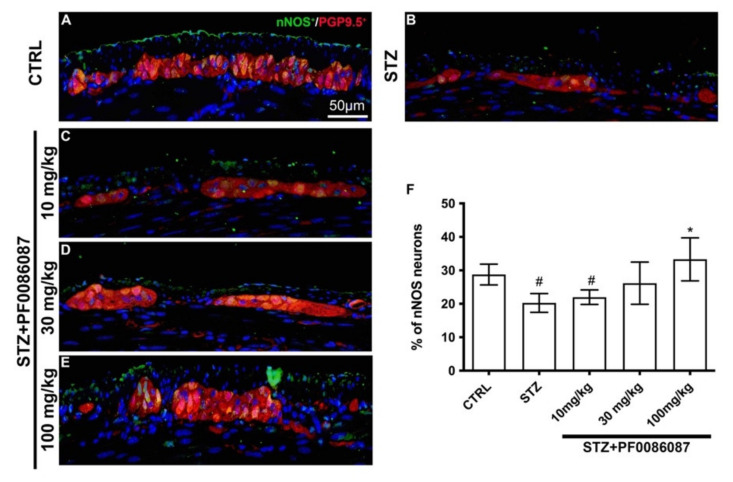
PGP9.5 and nNOS double labelling in the myenteric ganglia of mice. (**A**–**E**): Micrographs are representative, at 40× magnification, of PGP9.5 positive neurons (in red) and nNOS positive neurons (in green). DAPI labelling stained nuclei (blue). All the nNOS positive neurons are also PGP9.5 positive. Scale bar = 50 µm. (**F**): quantification of nNOS neurons. The nNOS neurons are expressed as a percentage of the PGP9.5 neuron number (nNOS/PGP9.5 ratio). One-way ANOVA test, significance of difference # *p* < 0.05 vs. CTRL; * *p* < 0.05 vs. STZ. STZ induced a significant decrease in nNOS/PGP9.5 ratio compared to controls. PF0086087 administration at 100 mg/kg was able to preserve NOS neuronal expression.

In STZ-induced mice, a significant decrease in nNOS/PGP9.5 ratio was observed, compared to controls (Figure 7, panels A, B and F). The administration of PF0086087 at 100 mg/kg prevented the STZ-induced effect upon the nNOS neuron subpopulation (Figure 7 panels B, E and F).

**Figure 8:**
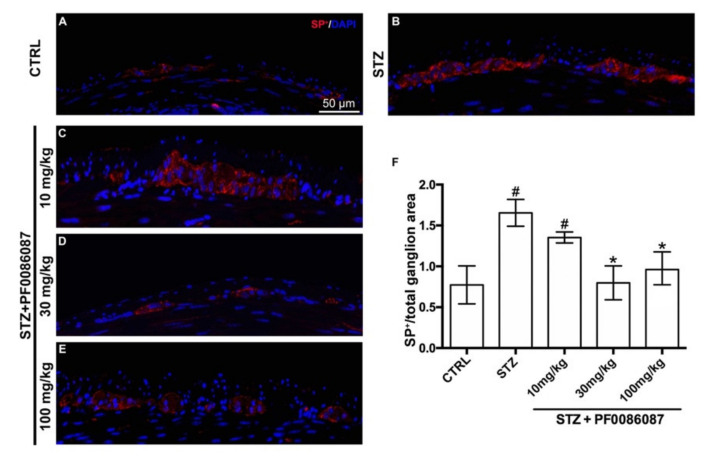
SP labelling in the myenteric ganglia of mice. (**A**–**E**): Micrographs are representative, at 40× magnification, of SP positive expression (in red). DAPI labelling stained nuclei (blue). Scale bar = 50 µm. (**F**): densitometric analysis of SP positive area per ganglion. Kruskal–Wallis test, significance of difference # *p* < 0.01 vs. CTRL; * *p* < 0.01 vs. STZ. A significant increase in SP nerve fibres was revealed in STZ-induced mice. PF0086087 administration at 30 and 100 mg/kg restored SP signal to the level of controls.

As SP is one of the most frequent initiators of neurogenic inflammation [27], its expression and density (fluorescence intensity) were measured in the myenteric ganglia of different experimental groups. A significant increase in the signal of SP nerve fibres was revealed in STZ-induced mice compared with controls (Figure 8, panels A, B and F); the histamine H_3_ receptor antagonist at 30 and 100 mg/kg was able to counteract this alteration, restoring SP signal to the level of controls (Figure 8, panels B, D, E and F).

**Figure 9:**
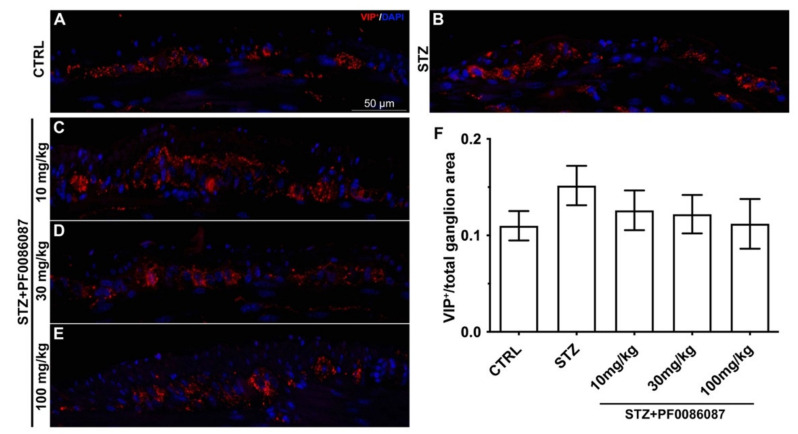
VIP labelling in the myenteric ganglia of mice. (**A**–**E**): Micrographs are representative, at 40× magnification, of VIP positive expression (in red). DAPI labelling stained nuclei (blue). VIP labelling was detected as small granules located within the myenteric plexus. Scale bar = 50 µm. (**F**): densitometric analysis of VIP positive area per ganglion. Kruskal–Wallis test was applied. No changes were revealed in VIP positive nerve fibres among the myenteric ganglia of different groups.

No significant changes were observed in either the density or fluorescence intensity of VIP nerve fibres among the myenteric plexus of different animal groups (Figure 9).

## 4. Discussion

The data we report herein indicate that the histaminergic system, and more specifically the antagonism of the peripheral histamine H_3_ receptor, may play a role in preventing diabetes-induced gastrointestinal complications.

Along with the other complications, diabetic neuropathy causes alterations in the sympathetic and parasympathetic nervous systems, leading to GI symptoms. GI functions are regulated by a specific and independent system, known as the enteric nervous system (ENS), embedded in the gastrointestinal tract wall [28]. The ENS consists of a complex network of neurons and enteric glial cells (EGCs), which bi-directionally communicate with enteroendocrine cells, other epithelial cells, blood vessels, and immune effector cells [29], mast cells included. In particular, mast cells, the primary endogenous source of histamine, exert excitatory effects on human submucous neurons, creating a functional axis with the ENS in the human intestine [30]. Enterochromaffin-like (ECL) endocrine cells, mast cells and neurons express the histamine H_3_ receptor. Although Sander L.E. et al. (2006), by immunostaining, revealed that histamine H_3_ receptor is absent in the healthy human ENS [31], the functional/pharmacological evaluation by Breunig E. et al. (2007) demonstrated that the histamine H_3_ receptor mediates excitatory effect in human submucous plexus [32]. Our results further support these findings, as follows: PF0086087, a CNS-sparing histamine H_3_ receptor antagonist, with low penetration to the blood–brain barrier ([Brain](free)/[plasma](free) ratio = 0.1 vs. 1.6 for PF008608 or the fully brain-penetrant reference antagonist, respectively, both administered to rats after 6 h iv infusion [24]), acting on the myenteric plexus of the distal colon, preserved the functional state of its glandular epithelium, as well as the excitatory (SP) and inhibitory (nNOS) components of the myenteric plexus, negatively affected by STZ exposure. Due to its pharmacodynamics, the effects observed for PF008608 can be ascribed to the only histamine H3 receptor binding. Indeed, in the study by Lunn G. et al. (2012), PF008608 showed a human histamine H3 receptor binding affinity and functional *K*_i_ < 10 nM (ranging from 0.832 nM for the cell-based functional response up to 9.6 nM for the binding assay in the presence of the ^3^H-N-alpha-methyl histamine agonist). Nevertheless, the *K*_i_s of PF-0868087 for the histamine H_1_, H_2_, and H_4_ receptors were >4 µM. The authors also confirmed no significant other pharmacological targets—the *K*_i_ measured for the sigma receptor and the hERG ion channel were 3.9 µM and >40 µM, respectively.

The STZ model is reminiscent of type 1 diabetes mellitus, inducing hyperglycaemia by damaging pancreatic ß-cells [33]. Moreover, this model has been previously reported to raise the level of histamine in different tissues [34,35], intestine included [36]. Due to the hyperglycaemic status, the extrinsic sympathetic supply is more sensitive to the ENS, via the coeliac and superior mesenteric ganglia than the superior cervical ganglion [37]. This condition causes marked structural remodelling of the gastrointestinal tract wall and its neuronal support leading to alteration of GI function [29]. In our study, the general morphology of the distal colon was preserved in STZ-induced mice, with no relevant signs of degeneration, except for the presence of oedema in the submucosa, prevented by the highest doses of the treatment. Nonetheless, increased production of mucins, revealed with the PAS reaction, in the STZ-induced animals compared to controls. This biochemical change was previously in STZ-induced diabetic rats [38], and by Domenec A et al. (2011), who reported that the crypts of diabetic RIP-I⁄hIFNb transgenic mice tend to contain more mucus in the lumina [39]. The observed over-production of mucin may be due, in part, to goblet cell hyperplasia in response to colon dysmotility to accelerate the replenishment of the mucus layer. In light of these considerations, it appears reasonable that the protective effect provided by PF0086087 on the neurochemical changes of the enteric neurons could counteract colon dysmotility, preventing goblet cell hyperplasia.

Many studies indicated that the different neuronal subpopulations of the GI tract are differentially susceptible to the development of neuropathy following hyperglycaemia. Therefore, to study the STZ-induced neuronal alteration at the myenteric plexus, we investigated the expression of SP (released by excitatory neurons), NO and VIP (released by inhibitory neurons). Pathological changes in these pathways led to detrimental effects on motor control with delayed emptying, impaired accommodation, and gastric dysrhythmia [40]. Being involved in both neurotransmission and immunomodulation, SP and VIP are known to initiate neurogenic transmission [27]. We could not observe any variations in the density or fluorescence intensity of VIP nerve fibres among the different animal groups, while increased SP immunoreactivity was observed in the myenteric ganglia of STZ-treated mice.

The undecapeptide SP, belonging to the family of the tachykinin/neurokinin, is a small neuropeptide acting as a neurotransmitter and neuromodulator. SP is widely expressed in different tracts of ENS, including the oesophagus, stomach, duodenum and colon [41], across all intestinal layers of the submucosa and myenteric plexus [42]. Although primarily linked to the modulation of proper sensory and nociceptive perception, SP further modulates the intestine’s immunological, vascular, and motor phenomena and exerts functions as a pro-inflammatory molecule [43,44]. Dysregulation in SP expression at the GI level has been described in diabetic conditions, as follows: SP content in the rectal mucosa of diabetic patients was significantly higher than that of non-diabetic controls, and neuropeptide levels were more than double in diabetics with constipation [45]. Similarly, SP immunoreactivity was observed in the GI tract of different diabetic animal models [46] and mice exposed to a high-fat diet [47]. In our hands, we observed increased SP immunoreactivity in the myenteric ganglia of STZ-treated mice, a state dose-dependently ameliorated by the histamine H_3_ receptor antagonist, PF0086087. The use of H_3_ receptors antagonists have been described to effectively reduce allodynia and hyperalgesia in neuropathic and inflammatory pain [6,48,49,50], and evidence suggests that SP released from peripheral sensory neurons is involved in both inflammatory and neuropathic pain [51]. Accordingly, in our model, the histamine H_3_ receptor antagonist, PF0086087, designed not to cross the blood–brain barrier, acting on the myenteric plexus of the distal colon, could reduce SP immunoreactivity.

The increased tachykininergic tone in the enteric glia appears to be involved in the onset of enteric motor alterations [47]. NO generated by enteric neurons is known to regulate the non-adrenergic non-cholinergic relaxation of smooth muscle, thus modulating colonic motility [52]. The selective loss of nNOS in humans’ diabetic colon [53] has been reported. However, according to Cellek’s biphasic model, the nitrergic neurons of the GI tract undergo a two-step degenerative process during diabetes, as follows: in the first phase, nNOS expression decreases without neuronal loss, while, in the second phase, the nitrergic neurons activate apoptotic processes [54]. In our study, two weeks after diabetes induction by STZ, we observed a reduction in the expression of the nNOS positive neurons, while the pan-neuronal marker PGP9.5 remained unchanged. The observed prominent nitrergic dysfunction without neuronal loss in the myenteric plexus of the distal colon is reminiscent of the first phase of Cellek’s biphasic model, before the so defined “point of no return” [54]. The preventive administration of PF0086087, dose-dependently, counteracted the effects of STZ on nNOS expression, at least removing the “point of no return” and, consequently, the occurrence of the neuronal loss in the myenteric plexus.

## 5. Conclusions

In conclusion, our data indicate that the histaminergic system plays a vital role in the onset of hyperglycaemia health complications and that the use of the CNS-sparing histamine H_3_ receptor may be an essential tool in the prevention of nitrergic dysfunction in the myenteric plexus of the distal colon and, therefore, in diabetes-induced GI complications.

## Figures and Tables

**Table 1:** Primary and secondary antisera used in immunohistochemistry.

Antigen	Species	Source	Concentration
**Primary Antisera**			
nNOS	Rabbit	Millipore (Bedford, MA, USA)	1:2000
PGP9.5	Mouse	Santa Cruz Biotech (Santa Cruz, CA, USA)	1:500
SP	Rat	Santa Cruz Biotech (Santa Cruz, CA, USA)	1:500
VIP	Mouse	Santa Cruz Biotech (Santa Cruz, CA, USA)	1:200
**Secondary Antisera**			
Alexa Fluor 594	Mouse	Jackson ImmunoResearch (Ely, Cambridgeshire, UK)	1:175
Alexa Fluor 488	Rabbit	Jackson ImmunoResearch (Ely, Cambridgeshire, UK)	1:175

nNOS = neuronal nitric oxide synthase; PGP 9.5 = protein gene product 9.5; SP = Substance P; VIP = vasoactive intestinal peptide.

## Data Availability

Data are contained within the article. Raw data supporting the findings are available from the corresponding authors upon reasonable request.
